# Healthcare managers’ experiences of technostress and the actions they take to handle it – a critical incident analysis

**DOI:** 10.1186/s12911-020-01261-4

**Published:** 2020-09-25

**Authors:** Magdalena Stadin, Maria Nordin, Eleonor I. Fransson, Anders Broström

**Affiliations:** 1 School of Health and Welfare, Jönköping University, P.O. Box 1026, SE-551 11 Jönköping, Sweden; 2 Department of Information Technology, Uppsala University, Uppsala, Sweden; 3 Department of Psychology, Umeå University, Umeå, Sweden; 4 Department of Clinical Neurophysiology, Linköping University Hospital, Linköping, Sweden

**Keywords:** Technostress, ICT demands, Digitalisation, eHealth, Occupational health, Managers

## Abstract

**Background:**

Healthcare managers, in comparison with other healthcare professionals, have an increased likelihood of experiencing technostress at work. Since knowledge about the causes and severity of technostress and about the strategies healthcare managers use to handle it is limited, the aim of this study was to describe their experience of technostress and the actions they employ to address it.

**Methods:**

An explorative design based on the critical incident technique was used. In total, 20 healthcare managers (10 women, 10 men) from four hospitals in two county councils in Sweden were purposively selected according to professional background, hierarchical management position, control span, time in the management position, and sex. Semi-structured interviews with regard to critical incidents and actions taken to handle technostress were conducted.

**Results:**

Healthcare managers’ experiences of technostress (*n* = 279) were categorised related to three main areas. These involved ‘negative aspects of digital communication’ (e.g. high workload, invasion of private life, and negative feelings related to digital communication), ‘poor user experience of ICT systems (such as illogicality of the ICT system, time-consuming ICT system, or malfunctioning ICT system) and ‘needs to improve organisational resources’ (e.g. needs associated with digital literacy, user influence and distribution of work and ICT systems). Actions taken to handle technostress (*n*=196) were described relating to three main areas involving ‘culture, norms and social support’ (such as good email culture, and co-worker support), ‘individual resources’ (e.g. individual strategies and competence) and ‘organisational resources’ (such as IT-related assistance and support).

**Conclusions:**

Healthcare managers described negative aspects of digital communication, poor user experience of ICT systems, and lack of organisational resources as potential technostress creators. These problems were handled by taking action related to culture, norms and social support, and individual as well as organisational resources. All these features, along with consideration of healthcare managers’ job demands and resources in general, should be incorporated into actions monitored by healthcare organisations to improve or maintain a sustainable digitalised environment for healthcare managers.

**Trial registration:**

Regional Ethics Board in Linköping #2017/597–31. Registered 20 March 2018. URL not available.

## Background

The digital work environment refers to the possibilities and problems related to physical, psychosocial and cognitive dimensions, as a result of the digitalisation of the supporting systems and tools at work [1]. This study encounters the psychosocial dimension of the digital work environment which is characterised by work in different information and communication technology (ICT) systems, including communication systems (e.g. email and social media), administrative systems (such electronic medical records and billing systems), video conference systems, and business intelligence systems. The digital work environment facilitates many work duties, contributes to increased efficiency and has great potential for organisational development [2, 3]. At the same time, there are many technostress creators related to the use of ICT at work that can contribute to technostress [4, 5]. Technostress creators that can be divided in the dimensions ‘techno-overload’, ‘techno-complexity’, ‘techno-insecurity’, ‘techno-uncertainty’, ‘techno-invasion’, ‘techno-unreliability’, ‘stress in human-machine interaction’, ‘technological workplace surveillance’ [4] (Additional file 1: Appendix 1). In the literature, several other concepts related to the digital work environment have been used, for example ‘ICT demands’, telepressure’ and ‘stress related to information systems’, which include some of the dimensions of the technostress concept [6–8].

Managers in general, and especially healthcare managers have been found to have higher rates of technostress operationalised as ICT demands, than other occupational groups [9]. Physicians in management positions have also been observed to have higher rates of technostress, operationalised as ‘stress related to information systems’, based on longitudinal data of over 1000 physicians followed over nine-year period [8]. Previous studies about the digitalised work environment for healthcare professionals in general have mainly focused on the implementation and use of electronic medical records [8, 10–14]. Features such as information overload, poor user experience of ICT systems, invisible work, and changes in job roles and professional status have been observed [8, 10–14]. However, the occurrence of technostress in healthcare managers is likely to be related to features other than the work with electronic medical records, since healthcare managers seldom use electronic medical records [8]. Aspects such as a high overall workload and many administrative work duties channelled via ICT, but little time to manage these work duties, might be part of the explanation for healthcare managers reporting high rates of technostress [8].

Technostress has been found to be associated with suboptimal self-rated health, cognitive disturbances, sleep disturbances and symptoms of burnout, etc. [6, 15–17]. However, the negative impact technostress might have on health could be balanced by adequate resources [18]. According to the Job-Demands-Resources model, resources of different kinds (e.g. individual, psychological, organisational) buffer the relationship between job demands (such as technostress creators) and health [18]. Potential resources in the digitalised work environment are partly the same as in the work environment in general, such as job control (e.g. influence on the working situation and education), reward (such as financial reward, esteem reward, job security and good career prospects) and social support from co-workers and executives [6, 19, 20]. In addition, there is some knowledge about resources specifically directed towards the digitalised work environment, sometimes referred to as ‘technostress inhibitors’ or ‘ICT resources’ [4, 21]. These involve, for instance, organisational facilitation of increased digital literacy, good IT support, and user influence (‘user’ refers to the individual who uses the ICT system) in the development or upgrading of ICT [4, 21]. The idea of user integration is also in line with the concept of sociotechnical systems. This theory proposes that joint optimisation with regard to the use of ICT systems, productivity and sustainable work is easier to accomplish if system developers and users interact [22]. By doing this, both technical and social (e.g. work environmental) aspects are incorporated into the system development of ICT systems [22]. Knowledge about the causes and severity of technostress among healthcare managers is limited. More knowledge about the digital work environment and the work environment in general among those in healthcare management positions is warranted. Potential barriers with respect to a sustainable digital work environment need to be identified, as well as strategies for handling technostress creators. Therefore, it is also of interest to describe healthcare managers’ organisational needs for an improved digital work environment.

### The Swedish healthcare system

All Swedish residents have the right to use public health care services, including primary health care service [21]. The responsibility of the health care service provision in Sweden is divided between the government, the 21 county councils and the 290 municipalities. The county councils provide most of the healthcare services in Sweden. In addition, there are private care alternatives (mostly in primary care). These have agreements with the county councils, that ensures the same compensation as the public health care providers and that the patients pays the same fee as in public health care services. The healthcare services (including both public and private actors) are mainly financed through regional taxation. Additionally, the county councils and municipalities receive some pre-directed financial support by the government [23]. All health care services in Sweden use electronic medical records and all patients over the age of 16 have access to their electronic medical records online. A patient contact is required for the health care professional to use the electronic medical record. Hence, healthcare professionals with no patient contact, does not work in the electronic medical record system. All public hospitals in Sweden have a central IT-support. Some clinics have specific ICT systems designed for their type of care. For these ICT systems, the specific IT support can be located at the clinic, in Sweden, or abroad.

### Aim and research questions

It is previously known that healthcare managers have high exposure to technostress [8, 9]. However, since quantitative studies are restricted to specific scales of technostress, it is warranted to complement the quantitative studies with a qualitative approach that describes the cause and of severity of technostress among healthcare managers. Even though there is some knowledge about technostress inhibitors [4, 21], the knowledge about healthcare managers resources with regard to the digital work environment is scarce. A deeper understanding of how health care managers experience their digital work environment and which actions they take to handle specific incidents related to this part of their work environment is needed. Consequently, the aim of this study was to describe healthcare managers’ experience of technostress and their actions for handling it. The following research questions were raised.

RQ1) In what manner and to what extent do healthcare managers experience technostress?

RQ2) What are the actions employed by healthcare managers to handle technostress?

RQ3) How successful are the actions employed by healthcare managers to handle technostress?

## Methods

### Design

A qualitative study design based on the critical incident technique was used in this study. The technique aims to identify critical experiences and actions taken for a specific purpose [24–26]. Historically, the critical incident technique was developed in the US Air Force aviation programme, during World War II, to reduce critical mistakes made by air force pilots [24]. Since then, the technique has been modified to fit additional contexts and research disciplines, such as health and caring science [25, 26]. In its current use, the technique has been described as a flexible qualitative method, useful in solving practical problems [25, 26]. The critical incident technique is an inductive method, and an unbiased search was done in the data material for the phenomenon without having assumed in advance that certain characteristics exist [26].

### Sampling and informants

Purposive and selective sampling was used in order to incorporate a rich variety of experiences and actions with regard to technostress among healthcare managers. The sample included representatives with different professional backgrounds, hierarchical management positions, control span (i.e. the number of subordinates under a healthcare manager), time in the management position, and sex. Informants in the study sample were recruited from four public hospitals in two middle-sized county councils in Sweden, and consisted of 20 healthcare managers (10 men, 10 women) in various organisational hierarchal management positions (Table 1). The included hospitals had between 1300 and 6000 employees, and the range of hospital beds were between 230 and 600 hospital beds. With regard to the digital work environment, the informants was working in different ICT systems, including communication systems (e.g. email and social media), administrative systems (e.g. billing systems), video conference systems, and business intelligence systems. However, since most of the informants did not have patient contacts, most of them did not work in the electronic medical records. If they did, it was mostly related to deviation reports etc.

**Table 1:** Socio-demographic characteristics of healthcare managers (*n* = 20)

*Sex*	
Men	10
Women	10
*Professional background*	
Physician	5
Nurse	4
Biomedicals/Pharmaceutics	4
Occupational therapist	2
Social worker	2
Other background	3
*Control span*	
< 50	8
50–100	5
> 100–300	5
≈ 2000	2
*Time as a healthcare manager*	
1–5 years	5
6–10 years	3
11–19 years	3
20–40 years	9

Note. ‘Control span’ refers to the number of subordinates under a healthcare manager; ‘Other background’ refers to a professional background with only one representative

### Procedure

Initially, potential informants were identified by four researchers (AB, BAG, DM, EF) who have a professional clinical background and/or previous cooperation with hospitals from which the informants were recruited. The first author invited the potential informants by email, including an information letter and a folder for informed consent, which were compulsory for participation. In the next step, based on the purposive selection criteria, new names were gathered either from the informants, or by contacts with persons in management positions at the in hospitals in the county councils. Healthcare managers who were interested in participating replied to this email. This process continued until 20 informants had been interviewed. A few days before the interviews, all informants received a reminder including short information and suggestions of how to prepare for their interview. All interviews were conducted by the first author. Most of the interviews (18 interviews) were conducted at the workplace of the informants, one was conducted at the workplace of the first author, and one was conducted via telephone, in line with the request of the informant. The mean duration of the interviews was approximately 39 min (range 25–70 min).

### Data collection

Information about experiences and actions was collected by semi-structured interviews [27]. An interview guide was developed by all co-authors (Table 2), in accordance with an interview guide applicable to the critical incident technique [26]. The interview guide was tested in a pilot interview and then slightly modified by the researchers after evaluating feedback from the pilot informant and data gathered in the interview. The informants were asked to describe critical incidents with regard to experiences when the work with ICT systems had been demanding, problematic or frustrating in some way, and how they had handled these situations. Follow-up questions were asked in relation to the response of the informant, in order to obtain a complete description of how the critical incidents were experienced and what actions were used to handle them (Table 2). The interviews were audio recorded and transcribed verbatim continuously (the first author transcribed half of the data material, and the rest was transcribed by an external transcriber).

**Table 2:** Interview guide applicable to the critical incident technique

*Background question:*	
✓ Please describe in what way you are working with ICT systems in your daily work	
*Main questions:*	
✓ Please tell me about different incidents and examples of when the work with ICT systems has been demanding, problematic or frustrating in some way ✓ Thinking about each incident or situation separately, tell me more about how you handled them	
*Potential follow-up questions:*	
✓ Where did the critical incident happen? ✓ Can you give a detailed description of what happened? ✓ Why was the incident critical for you? ✓ What did you do in connection with the incident? ✓ What was your mindset during the incident? ✓ What were your thoughts during and after the incident? ✓ What were your feelings during and after the incident? ✓ What did you find was the most demanding aspect of the incident? ✓ What has this incident meant to you since?	

### Data analysis

To strengthen creditability, the transcribed data material was initially read through by the authors and then discussed among the co-authors with regard to the aim of the study to identify main areas. Critical incidents with regard to experiences of technostress, and the actions taken to handle these incidents, were identified and carefully compared, abstracted and labelled into subcategories, categories and main areas, in line with previous suggestions for this technique [26]. The data analysis process took place simultaneously with the data collection, and all co-authors were involved and contributed to all parts of the process (including competence in using the critical incident technique and theoretical knowledge of healthcare science, occupational health, public health, psychology and epidemiology) to further improve creditability.

## Results

Critical incidents with regard to technostress creators (*n* = 279) were identified and categorised into the main areas: ‘negative aspects of digital communication’, ‘poor user experience of ICT systems’ and ‘needs to improve organisational resources’ (Table 3). Actions to handle technostress (*n* = 196) were categorised into the main areas ‘culture, norms and social support’, ‘individual resources’, and ‘organisational resources’ (Table 4). The association between critical incidents and actions are shown in Table 5. Citations that illustrate critical incidents and actions are shown in Additional file 1 : Appendix 2 and Appendix 3 respectively.

**Table 3:** Healthcare managers’ experiences of technostress, explored by critical incident technique

Main areas	Categories	Subcategory (number of incidents)
Negative aspects of Digital communication	High workload	High number of digital messages (39 incidents)
		Redundant digital messages (18 incidents)
		Demands for rapid replies to digital messages (13 incidents)
	Invasion of private life	Limitless digital communication management (11 incidents)
		Distressing messages after working hours (5 incidents)
	Negative feelings	Fear of missed information in the inbox (11 incidents)
		Misunderstandings or frustration in digital communication (6 incidents)
Poor user experience	Time consuming ICT systems	Illogical ICT systems (15 incidents)
		Sequence-ordered ICT systems (5 incidents)
		Login frustration (12 incidents)
		Slow response time of ICT (6 incidents)
	Malfunctioning/disturbing ICT systems	Technical struggle (22 incidents)
		Disturbances from notifications (12 incidents)
		Updates that require action (8 incidents)
Needs to improve organisational resources	Facilitating digital literacy	Lack of general digital literacy (10 incidents)
		Lack of literacy in ICT systems used irregularly (9 incidents)
		Need for practical training in ICT (14 incidents)
	Facilitating user influence	Need for increased user influence (12 incidents)
		Need for increased terminology fit between ICT systems and healthcare (10 incidents)
		Need for increased use of new digital solutions (7 incidents)
	Redistribution of work and ICT systems	Need for increased administrative support (17 incidents)
		Need for redistribution of ICT systems (12 incidents)
		Need for strengthened back-up routines for system breakdowns (5 incidents)

**Table 4:** Healthcare managers’ actions related to technostress, explored using critical incident technique

Main areas	Categories	Subcategory (number of actions)
Culture, norms and social support	Good email culture	Efficient digital communication management (12 actions)
		Meetings instead of emails (8 actions)
		Communication about digital communication with co-workers (15 actions)
	Co-worker support	Situation-based co-worker support (8 actions)
		Reliance on co-worker’s digital literacy (7 actions)
		Supporting each other during system failure (5 actions)
Individual resources	Individual strategies	Routine and structure (16 actions)
		Flexible in replying to digital communication (13 actions)
		Using digital solutions (8 actions)
		Using separate digital device for work and private life (6 actions)
	Individual competence	Digital literacy (10 actions)
		Learning by doing (14 actions)
		Preparation (12 actions)
		Improvisation (9 actions)
		Confident attitude (7 actions)
Organisational resources	Support and assistance	Good IT support (18 actions)
		Back-up routines (8 actions)
		Administrative support (20 actions)

**Table 5:**
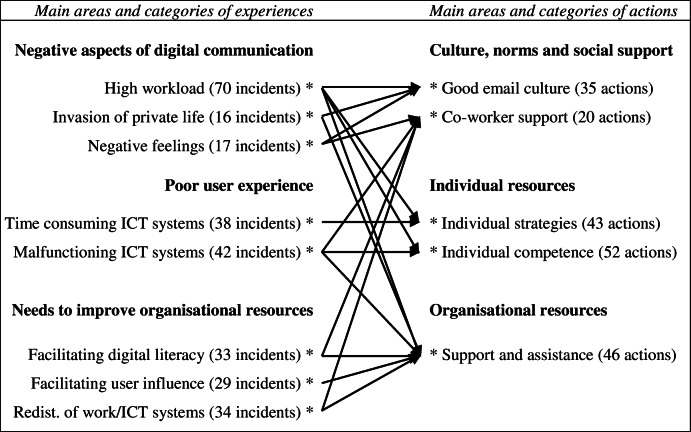
Association between healthcare managers experiences and actions related to technostress

### Experiences of technostress among healthcare managers

#### Negative aspects of digital communication

Negative aspects of digital communication involved high workload, which referred to a large number of incoming messages (e.g. emails), including demands to reply rapidly (Table 3; Additional file 1: Appendix 2). Some incoming digital messages, such as mass emails and copies, were described as redundant. The managers stated that digital communication invaded private life, which was stressful. Some managers reported that it was more or less expected by co-workers that they should be available via ICT outside working hours. Distressing messages received after working hours were described as very disturbing. This was exemplified, for instance, by an incident that referred to a threat towards a physician via email (Additional file 1: Appendix 2). Digital communication involved frustration, anger and misunderstandings, which evoked negative feelings.

#### Poor user experience of ICT systems

Poor user experience of ICT systems referred to ICT systems that were time-consuming for different reasons, and additionally, malfunctioning and disturbing ICT systems (Table 3; Additional file 1: Appendix 2). The healthcare managers stated that they were working in front of a computer during most of the working day, and that they used many different ICT systems (such as communication systems, video conference systems, administrative systems, and business intelligence systems). Consequently, poor user experience of certain ICT systems was described as very frustrating, since they were exposed to it so frequently. The experience of unnecessary time consumption in using ICT systems was related to illogical, non-intuitive and sequence-ordered systems (the latter was troublesome if the commands were unintentionally conducted in the wrong order). Additionally, unnecessary time consumption was related to login aspects in different ICT systems, as well as slow system response. Malfunctioning or disturbing ICT systems referred to incidents related to technical struggle (e.g. during a video conference meeting). Moreover, disturbances from different types of notifications were reported to break concentration. Undesired updates of ICT systems were likewise perceived as disturbing, partly since they required an update in literacy to manage the system.

#### Needs to improve organisational resources

Needs to improve organisational resources referred to needs related to digital literacy facilitation, user influence, and redistribution of work and ICT systems (Table 3; Additional file 1: Appendix 2). Lack of digital literacy was experienced as a barrier to conduct ICT-related working duties for some healthcare managers. Embarrassment related to digital literacy was also described when they experienced that they did not live up to the societal expectation of digital literacy and skills. Needs relating to digital literacy facilitation involved, for instance, practical training in managing ICT, preferably with a mentor available for questions and support. Needs relating to user influence referred, for example, to increased user influence in the development or upgrading of an ICT system and increased terminology fit between ICT and healthcare. An example of this is that the established term ‘referral’ was replaced with ‘care request’ in the electronic medical records. Redistribution of work and ICT systems referred, for instance, to increased need for administrative support. Healthcare managers declared that it was hard to find enough time for administrative work duties, such as replying to emails, especially during days when most of the working time was spent in meetings, and they consequently stated a need for increased access to administrative support.

### Actions related to technostress among healthcare managers

#### Culture, norms and social support

Culture, norms and social support referred to a good email culture and co-worker support, respectively (Table 4; Additional file 1: Appendix 3). A good email culture was described as efficient digital communication, which included strategies for providing clear and purposive information that was easy to interpret. Additionally, it included strategies for reducing the number of digital messages, such as avoidance of sending copies, mass emails and ‘replying-to-all’, but also included discussing certain questions in meetings instead of via a digital channel. Co-worker support referred to aspects such as reliance on a co-worker’s digital literacy, to sitting together with a co-worker during a video conference meeting, or to co-workers supporting each other during system failures.

#### Individual resources

Individual resources included individual strategies and individual competence (Table 4; Additional file 1: Appendix 3). They also encompassed routine and structure; for instance, scheduling time for administrative work duties channelled via ICT systems. Other examples of individual strategies were using separate hardware for work and private life. Individual resources referred to digital literacy, i.e. having the competence to manage ICT systems adequately. Individual resources also involved different strategies, such as learning by doing, preparation (e.g. before a PowerPoint presentation), improvisation, and having a confident attitude towards potential technostress creators.

#### Organisational resources

Organisational resources involved IT-related support and assistance (Table 4). Accessible, efficient and helpful IT support was referred to as ‘good’ IT support. Back-up routines for system breakdown were also described as a resource. The possibility to delegate administrative job duties, such as managing invoices, replying to emails, and preparing a PowerPoint presentation to healthcare administrators, were experienced as very helpful, firstly as they decreased the managers’ workload, but also since the administrators were considered better qualified to carry out such work duties.

## Discussion

Healthcare managers’ experience of technostress and their actions to handle technostress were described in this study. High rates of technostress have been observed in healthcare managers in quantitative studies previously [8, 9]. Technostress and indications of non-sustainable digital work environments have also been reported by other healthcare professionals previously [8, 10–14]. However, this is the first study that explores healthcare managers’ own experience of technostress and, in particular, how they handle it. Technostress creators were categorised as ‘negative aspects of digital communication’, ‘poor user experience of ICT systems, and ‘lack of organisational resources’ (e.g. lack of digital literacy facilitation), findings which share similarities with previous studies [4, 21, 28]. However, to understand the potential reasons why healthcare managers have previously been observed to have an increased likelihood of technostress [8, 9], it is necessary to consider their work demands in general. High overall job demands and considerable time spent in meetings can be considered barriers to a sustainable digital work environment, since there is not sufficient time for managing ICT-related administration and updating digital literacy. Consequently, the distribution of work duties from an organisational perspective should be considered in efforts to establish a sustainable digital work environment for healthcare managers, and professionals in the healthcare organisation in general.

This study intended to describe technostress among healthcare managers from a broader perspective. However, there was an uneven representation with respect to the technostress creator dimensions presented in Additional file 1: Appendix 1. The healthcare managers mainly described critical incidents of technostress that can be related to the techno-overload and techno-invasion dimensions in of technostress creators [4, 5]. Experiences that can be related to techno-complexity, techno-uncertainty, and technological workplace surveillance dimensions were also observed. Whether any of the incidents can be related to the techno-insecurity dimension is uncertain. None of interviewed healthcare managers reported that they were afraid of losing their job due to lack of digital literacy, or due to artificial intelligence. Some healthcare managers rather described embarrassment related to lack of digital literacy and that they experienced that they did not live up to the societal expectation of digital literacy and skills. One healthcare manager even described that he intentionally had applied for a manager position in which there was sufficient access to administrative and IT-related support, to balance the lack of digital literacy that this healthcare managers experienced. Possibly, insufficient digital literacy is a bigger problem for healthcare managers on lower positions, who do not have the same amount of organisational resources in terms of administrative and IT-related support.

Resources of different sorts can balance the potentially negative influence that technostress has on health [4, 18, 21]. A number of different actions to handle potential technostress creators were identified in this study, and while some of these actions can be considered to be resources for a sustainable digital work environment, others can be seen rather as workarounds in a non-sustainable digital work environment. For instance, actions related to efficient digital communication and adequate digital literacy, can be considered to be resources, since they are solution focused. On the other hand, actions such as digital communication management before or after working hours, are workarounds, since the potential technostress creator are still present. However, to separate actions with regard to resources and workarounds is complex. For instance, working with alternative work duties (such as email reply) when an ICT system has slow response time to start-up, or during technical struggle on a video conference meeting, is on the one hand a useful way of spending the worktime. On the other hand, the potential technostress creator, in this case slow response time in ICT systems or technical struggle, would still be an issue. Alternatively, to sit together with a co-worker during a video conference meeting, or co-worker support during system failure, could both be an opportunity to learn from each other and simultaneously improve digital literacy. However, an imbalance in digital literacy can also result in one co-worker taking the full responsibility of the technological issues, and the other becoming dependent on this individual. Consequently, even though individuals may find workarounds to manage a non-sustainable digital work environment, the organisation should implement targeted actions that reduce or eliminate potential sources of technostress.

Poor user experience of certain ICT systems was experienced as a technostress creator, which is in line with previous findings [4, 29]. User experience is defined as all aspects of the user experience when interacting with the ICT systems, service, environment or facility [30]. Good user experience is characterised by an ICT system that is useful, usable, findable, credible, desirable, accessible and valuable [30]. Needs and actions to improve the user experience of ICT systems were related to user influence on ICT systems. Increased interaction between system developers and users (in this case healthcare managers), including incorporation of both technical and social work aspects, would also be beneficial for productivity and sustainable work, according to the concept of sociotechnical systems [22]. However, even though many healthcare managers stated that they had user influence to some extent in the development or upgrading of an ICT systems, the time lag between the feedback to the system developers and the implementation of the upgraded system was experienced as very long. For instance, one healthcare manager stated that the time lag could be at least a year, and a previous study has found that it takes approximately 3 years to upgrade an ICT system in a healthcare organisation [31] after feedback from users. Therefore, although increased user influence is an important aspect for developing ICT systems that provide a good user experience, strategies to reduce the time lag between user feedback and the actual upgrade should be acknowledged.

Descriptions of organisational needs corresponded well with the descriptions of organisational resources, as suspected (Table 5). A review of work-related stress among nurse managers showed that lack of, or inadequate resources were their main source of work-related stress [32], which points to the importance of adequate resources in the association between job demands and health [18]. Whether an aspect was described as an organisational need or an organisational resource was partly related to the manager’s position. For instance, healthcare managers in higher positions typically had greater access to administrative support from healthcare administrators, and described this as a huge resource, while healthcare managers in lower positions described a need for extended support from healthcare administrators. People in higher work positions also have access to more resources in general that can balance the potentially negative influence technostress might have on health [33].

Previous studies have identified a few desired organisational resources in the digitalised work environment, such as digital literacy facilitation, good IT support, and user influence, as well as practical training, evaluation and support when implementing a new ICT system in an organisation [4, 21, 29, 34]. These findings correspond very well with the organisational needs or resources mentioned in this study. In other words, there is a considerable amount of existing knowledge that identifies key organisational ways to enhance a healthy and sustainable digital work environment in healthcare and organisations in general. It could consequently be questioned why the digital work environment in healthcare is not more sustainable already. The answer to this question is complex. It may partly be related to the research-practise gap paradox (also termed as the knowing-doing-gap), that refers to the gap between the scientific knowledge and what knowledge that is implemented in organisations [35]. However, many of the healthcare managers in this study stated that they made great efforts to improve the digitalised work environment for their clinic, area of responsibility or healthcare organisation in general. Still, different kinds of barriers partly prevented them from improving the digital work environment more efficiently, such as long-lasting time lags in accomplishing changes, juridical regulations, difficulties in changing work routines, and budget restrictions. These aspects are in line with previous findings in implementation research in healthcare, that points out sufficient financial resources and time availability, combined with supportive leadership and social support from co-workers, as key components for successful implementation [36].

### Methodological considerations

#### Methodological rigor of the critical incident technique

With regard to the aim, critical incident technique is an appropriate method for this study, partly since it is a useful method of describing the cause and severity of a problem, but particularly since the method is solution focused [24, 26]. Henceforth, the methodological rigor will mainly be discussed according to four methodological components that are relevant to obtaining trustworthiness in qualitative research, i.e. credibility, confirmability, dependability and transferability [37]. The credibility in the study was strengthened by good preunderstanding of work-related stress with a focus on digitalisation as well as of the critical incident technique among the co-authors. Follow-up questions in line with previous recommendations [26], were asked, in order to obtain descriptions of the critical incidents and actions that were as rich as possible. Confirmability and were strengthened by several re-readings of the transcriptions and careful labelling and comparison of subcategories, categories and main areas, with input from all co-authors. The objective perspective in the interpretation of the data is possibly strengthened by the fact that the first author, who conducted all interviews and was mainly responsible for data analysis, does not have a professional clinical background in healthcare, and has consequently no personal preunderstanding of technostress in healthcare. Additionally, confirmability was improved by the rich data set, based on 279 reported incidents and 196 reported actions [25, 26]. To increase dependability as well as transferability, efforts were made to obtain a rich variation in the study sample with respect to different characteristics. Additionally, the time range for data collection was 6 months and covered interview appointments before and after the summer vacation leave, to reduce potential bias related to the time of the year.

#### Limitations

This study has also some limitations to announce. A threat towards credibility may be that the critical incident technique, like many other qualitative methods, is dependent on the informants’ memories, and there is consequently a risk of recall bias (i.e. incidents that happened a long time ago might be incorrectly recalled or even forgotten) [24, 26]. The informants might also have chosen to not share information about themselves that reflect them in a bad manner, due to social desirability reasons [25, 26]. In addition, since critical incident technique requires descriptions of individual perspectives, and not descriptions in general terms [24], some potential critical incidents might be lost, since it is, for instance, common in the Swedish language to use the pronoun “one” instead of “I” in self-referring descriptions [38]. When this happened, the first authors asked follow-up questions that referred to the individual perspective. Minor nuances in the quotes might also be lost in the translation from Swedish to English. No quantitative measure of the investigator triangulation, such as the K coefficient of inter-rater reliability, was calculated, which could be considered a limitation.

## Conclusion

Healthcare managers describe negative aspects of digital communication (such as receiving large numbers of emails), poor user experience of ICT systems (such as illogical and time-consuming ICT systems) and lack of organisational resources (e.g. a need for increased administrative support) as technostress creators. High overall job demand and considerable time spent in meetings can be considered barriers to a sustainable digital work environment, since there is not sufficient time for managing ICT-related administration and updating digital literacy. Strategies to handle technostress included aspects related to culture, norms and social support (e.g. support from co-workers and communication about the email culture), individual resources (such as digital literacy) and organisational resources (e.g. administrative support). While some of these strategies (regardless of which main area they were categorised into) may contribute to a healthy and sustainable digital work environment, others were seen rather as workarounds to cope with an unhealthy work environment. For instance, efforts to reduce the administrative burden placed on them via ICT systems by implementing guidelines on how to reduce unnecessary emails, and by delegating administrative work duties, may be sustainable strategies, since they ‘solve’ the problem. On the other hand, the strategy of replying to emails during the few minutes between two meetings is not sustainable since it will not reduce the administrative burden from a long-term perspective. Doing so would be more likely to limit the possibility of psychological recovery during work [39]. However, even though individuals may find workarounds to manage a non-sustainable digital work environment, the organisation should implement targeted actions that reduce or eliminate potential technostress creators. This study should preferably be complemented by a quantitative study measuring organisational and individual resources in healthcare managers, as well as studies that evaluates targeted actions to improve the digital work environment among healthcare managers.

## Supplementary information

**Additional file 1: Appendix 1.** Author illustration of different dimensions of technostress creators. **Appendix 2.** Healthcare managers’ experiences of technostress, explored by critical incident technique. **Appendix 3.** Healthcare managers’ actions related to technostress, explored using critical incident technique.

## Data Availability

The data set in this study is not publicly available. Anonymised data is available from the corresponding author on reasonable request, if adequate legal, professional, and ethical approval can be provided.

## Abbreviations

ICT: Information and Communication Technology
IT: Information Technology
