# Supplementary material for: Healthcare managers’ experiences of technostress and the actions they take to handle it – a critical incident analysis
Source: BMC Med Inform Decis Mak. 2020 Sep 25;20:244. doi: 10.1186/s12911-020-01261-4 (PMC7517792; doi:10.1186/s12911-020-01261-4)
Supplement: Supplementary file 1 — Additional file 1: Appendix 1. Author illustration of different dimensions of technostress creators. Appendix 2. Healthcare managers’ experiences of technostress, explored by critical incident technique. Appendix 3. Healthcare managers’ actions related to technostress, explored using critical incident technique. [file 12911_2020_1261_MOESM1_ESM.docx]

| **Appendix 1.** Author illustration of different dimensions of technostress creators | |
| --- | --- |
|  |  |
| Techno-overload | *High job demands in relation to ICT systems due to high work pace, frequent interruptions, multitasking, prolonged working time, expectations regarding response times in digital communication etc.* |
|  |  |
| Techno-complexity | *Certain ICT systems are highly complex and challenge the user’s concentration, qualification, feeling of control, which requires additional time in handling complexity* |
|  |  |
| Techno-insecurity | *Fear of job loss or of a degradation in status caused by the perception that ICT systems (e.g. robotic process automation) or more qualified employees will fully or partly replace the own job/position* |
|  |  |
| Techno-uncertainty | *A constant feeling of uncertainty and ambiguity caused by chronic digital transformation processes or by features of single ICT systems which foster constant change* |
|  |  |
| Techno-invasion | *Mobile devices enable high flexibility, which can blur boundaries between work and other life domains,*  *causing work-life-conflicts and impair work recovery* |
|  |  |
| Techno-unreliability | *Stress induced by breakdowns, technical errors, low usability, and poor user experience in certain ICT systems* |
|  |  |
| Stress in human-machine interaction | *Irritation because of unpredictability of robot or machine behavior or unspecific anxiety towards robots, high complexity of such systems (see techno-complexity)* |
|  |  |
| Technological workplace surveillance | *New ICT systems can enable a close monitoring of work performance, location and working times, which can provoke distrust and loss of control in the monitored employees* |
|  |  |

Note. This illustration is according to Ragu-Nathan et. al (2008); Dragano & Lunau (2020) (4, 5)

| **Appendix 2** Healthcare managers’ experiences of technostress, explored by critical incident technique | | | |
| --- | --- | --- | --- |
| Quotations: experiences described by healthcare managers | Subcategory (number of incidents) | Categories | Main areas |
|  |  |  |  |
| “I receive about 40 emails per day. I know this because I usually delete my emails after six months, and then it is very easy to see how many emails there are in total, and then divide it by 180 days […] That is a source of stress for me.” (Informant 2) | High number of digital messages (39 incidents) | High workload | Negative aspects of digital communication |
|  |  |  |  |
| “Then there is this so called “email trash”, i.e. when I receive emails as “cc”, but they are not addressed to me in person. I will never reply to an email sent to me in “cc”. I think that if a message is sent to me, it should also be addressed to me.” (Informant 18) | Redundant digital messages (18 incidents) |  |  |
|  |  |  |  |
| “People send me emails, and they call me and say, “Have you seen this email?” These situations become very stressful, so to speak. It feels like I am being hunted.” (Informant 18) | Demands for rapid replies to digital messages (13 incidents) |  |  |
|  |  |  |  |
| “It has been very much so that I work outside working hours with the email inbox, from home, at the breakfast table, and in bed before going to sleep, you know. I live with it all the time, and that is a source of stress for me.” (Informant 1) | Limitless digital communication management (11 incidents) | Invasion of private life |  |
|  |  |  |  |
| “I was setting my alarm clock, and then I just logged in and checked my email inbox for the sake of safety, about 11.30 p.m. Then there was an email about a threat towards a physician at my clinic, and that is serious. It is not good to end the day like that, when you are supposed to go to bed.” (Informant 2) | Distressing messages after working hours (5 incidents) |  |  |
|  |  |  |  |
| “It might be something important in there, that I haven’t seen […]. I had organised a meeting for my co-workers […]. Then my co-workers told me that the other participants never arrived. ‘So strange’, I thought, and found out that they had cancelled the meeting two hours earlier by email.” (Informant 5) | Fear of missed information in the inbox (11 incidents) | Negative feelings |  |
|  |  |  |  |
| “A co-worker had been crying because of how I had replied to this email. I did not understand why and then I re-read my reply, but still I did not understand what was wrong with this reply. This individual must have perceived my reply in a sense that was not in line with my intention.” (Informant 4) | Misunderstandings or frustration in digital communication (6 incidents) |  |  |
|  |  |  |  |
| “Then there is plenty of information on the screen, I mean the whole screen is full of information. I have learned that I must click in that box, and in that box, and in that box, then it will be correct, but there is no logic in it.” (Informant 5) | Illogical ICT systems (15 incidents) | Time consuming ICT systems | Poor user experience |
|  |  |  |  |
| “In this module, things need to be done in sequence, which can be done in the wrong order, and if that happens, the whole case will be locked. So, it is problematic that it is possible to do it wrong, that it must be done in a special way, in sequence. It is very time consuming.” (Informant 13) | Sequence-ordered ICT systems (5 incidents) |  |  |
|  |  |  |  |
| “What I can feel frustrated about every day is all the logins, which is compulsory in all ICT systems. Then I am suddenly thrown out of the system if I have not been active for a while […] Just in and out of the ICT system every single day.” (Informant 11) | Login frustration (12 incidents) |  |  |
|  |  |  |  |
| It can take up to five or six minutes to log into the ICT system when I come in the morning. I believe that is an unnecessarily long time […]. When I log in I want it to pop up immediately. (Informant 20) | Slow response time of ICT systems (6 incidents) |  |  |
|  |  |  |  |
| “I always feel worried when we are going to have a larger video conference. I feel worried that it will not work out properly. A few times, it has not been working out, and then we have used emergency solutions like calling on the telephone instead, but that is not a good solution.” (Informant 15) | Technical struggle (22 incidents) | Malfunctioning or disturbing ICT systems |  |
|  |  |  |  |
| “Several ICT systems are delivering notifications while I am working in front of the computer […]. Like in the electronic medical record, there is this green notification box that pops up in front of all other windows, and I cannot click away this box for several seconds. That is really annoying” (Informant 13) | Disturbances from notifications (12 incidents) |  |  |
|  |  |  |  |
| “There is one of these start menus where ‘my cases’ is always visible on the start menu, but after the update nothing is there anymore. It is empty when I open the program. Then I think, ‘Good, I do not have any cases’, but I do have cases, it is just that I must search for them” (Informant 14) | Updates that require action (8 incidents) |  |  |
|  |  |  |  |
| “Then, when I am supposed to show the presentation, I am not sure which socket I should put the USB in. I just want my presentation up on the screen. It stresses me out. I feel like, ‘Where is it?’ Simply, I feel stupid, and that is somewhat embarrassing.” (Informant 9) | Lack of general digital literacy (10 incidents) | Facilitating digital literacy | Needs to improve organisational resources |
|  |  |  |  |
| “Six months can pass without using this ICT system. Then after six months I suddenly have to use this system again, but then I have forgotten how to manage it.” (Informant 11) | Lack of literacy in ICT systems used irregularly (9 incidents) |  |  |
|  |  |  |  |
| “First, a review of the entire ICT system, and then to sit together with a person with good literacy in the ICT, to practise some parts. Because I like hands-on-training best, so to speak.” (Informant 20) | Need for practical training in ICT systems (14 incidents) |  |  |
|  |  |  |  |
| “I have been a part of a reference group when new information is about to be implemented, but I have experienced that I am almost used as a ‘cover’ for an implementation that has already been decided. Kind of ‘Look, we asked the users’, but no one really listens to me.” (Informant 13) | Need for increased user influence (12 incidents) | Facilitating user influence |  |
|  |  |  |  |
| “It is very strange how things are defined and renamed. I cannot understand why a referral must now be termed ‘care request’, just as an example […] Why new terminology? Every human knows what a referral is.” (Informant 2) | Need for increased terminology fit between ICT systems and healthcare (10 incidents) |  |  |
|  |  |  |  |
| “There is a big stress for me, that within healthcare we have outdated ICT that are not adapted to the expectations of clients in the rest of the society […]. I feel like what I send to clients, or what clients want from me, I cannot live up to those expectations.” (Informant 6) | Need for increased use of new digital solutions (7 incidents) |  |  |
|  |  |  |  |
| “There must be a manager’s signature on everything, so I have to do everything myself. I think that is a pity […]. I think that it is an unnecessary way of spending my working time as a manager […]. A healthcare administrator could do a lot of these tasks instead.” (Informant 5) | Need for increased administrative support (17 incidents) | Redistribution of work and ICT systems |  |
|  |  |  |  |
| “The county council have three different ICT systems for video conferences. I have stated several times that we cannot have different ICT systems for this […]. I have mentioned this issue several times, that we need to use the same ICT system.” (Informant 4) | Need for redistribution of ICT systems (12 incidents) |  |  |
|  |  |  |  |
| “What happens if there is a system breakdown when only half of the staff are working? […] Some test results must be delivered within 30 minutes, when there are patients who are hovering between life and death in the intensive- or emergency-care units. That is what we are playing with.” (Informant 7) | Need for strengthened back-up routines for system breakdowns (5 incidents) |  |  |
|  |  |  |  |

| **Appendix 3.** Healthcare managers’ actions related to technostress, explored using critical incident technique | | | |
| --- | --- | --- | --- |
| Quotations: actions described by healthcare managers | Subcategory (number of actions) | Categories | Main areas |
|  |  |  |  |
| “In healthcare, we are pretty good at not sending ‘Hello and thank you’ back and forth; instead we are efficient in our emailing […] We are rather disciplined by that in my management team, and not many mass emails are sent out from my team.” (informant 5) | Efficient digital communication management (12 actions) | Good email culture | Culture, norms and social support |
|  |  |  |  |
| “I had a meeting with my closest managers, and then they brought their current questions to this meeting. Such things would have resulted in a number of emails back and forth otherwise, but now we were gathered together and went through these questions instead.” (Informant 16) | Meetings instead of emails (8 actions) |  |  |
|  |  |  |  |
| “About emails, I have told my co-workers that I am like a goalkeeper, and that a lot of balls (metaphor for emails) are thrown at me, and I try to throw these balls back as fast as I can, but sometimes some balls roll off on the side. In that case, they can throw these balls back again.” (Informant 12) | Communication about digital communication with co-workers (15 actions) |  |  |
|  |  |  |  |
| “Thankfully, we have a medical-technical resource here, that I would go to as a first choice. If this person is not available, I have to deal with it myself. Then I contact either IT support, or I ask a co-worker who has faced the same problem previously and who can show me how to handle it.” (Informant 14) | Situation-based co-worker support (8 actions) | Co-worker support |  |
|  |  |  |  |
| “It is not unusual that we have video conferences between the different hospitals. Then I usually go to a co-worker’s office that is nearby […]. I go to someone else, because I know that ‘this person will fix this.’” (Informant 16) | Reliance on co-worker’s digital literacy (7 actions) |  |  |
|  |  |  |  |
| “Then, when we can finally start our video conference meeting, I usually say something positive, like ‘only ten minutes.’ Otherwise we would have been one hour behind due to the travelling, time that we now are saving […]. Nobody wants to be the person that delayed a meeting by ten minutes.” (Informant 1) | Supporting each other during system failure (5 actions) |  |  |
|  |  |  |  |
|  |  |  |  |
| “I need to have some kind of routine that makes me feel that I have control over the email situation. I am happy to come to work 30 minutes early, before everybody else, to manage the email. Then I feel that I have the day under control.” (Informant 3) | Routine and structure (16 actions) | Individual strategies | Individual resources |
|  |  |  |  |
| “I keep myself entertained, so to speak. You know, when I have signed out from work, I have a short walk to the car, then I can check my email while I am walking.” (Informant 10) | Flexible in replying to digital communication (13 actions) |  |  |
|  |  |  |  |
| “Very early in my post as a manager, I learned to remove the notification function in the computer, so it does not sound when I am receiving emails. Because that is really disturbing.” (Informant 19) | Using digital solutions (8 actions) |  |  |
|  |  |  |  |
| “I have actively chosen not to use the same Android for work and for private life, for a few reasons. One of these reasons is that even if I must keep some track of work when I am off, I want to have the possibility to turn the work off, and especially the email.” (Informant 19) | Using separate digital device for work and private life (6 actions) |  |  |
|  |  |  |  |
| “I am pretty IT proficient myself, so I am able to solve some problems on my own.” (Informant 20) | Digital literacy (10 actions) | Individual competence |  |
|  |  |  |  |
| “If there is a new ICT system, I am the kind of person who wants to go straight into the ICT system and click around. I am more of a trial-and-error person. That works well for me. I like self-instructing ICT best.” (Informant 6) | Learning by doing (14 actions) |  |  |
|  |  |  |  |
| “If I have done it (given a PowerPoint presentation) for myself once, I have no problem with it. It does not stress me out so much […]. So, preparation is an important factor for me.” (Informant 8) | Preparation (12 actions) |  |  |
|  |  |  |  |
| “Then I improvise, and I am good at it. I have solved many situations like that. I just speak freely instead, and I solve it somehow.” (Informant 16) | Improvisation (9 actions) |  |  |
|  |  |  |  |
| “Maybe I was stressed about it before, but somehow with experience, I feel that the world will not end today if I do not reply to this email.” (Informant 17) | Confident attitude (7 actions) |  |  |
|  |  |  |  |
|  |  |  |  |
| “In my current management position, we have an IT strategist at our department, who is very proficient when it comes to IT.” (Informant 17) | Good IT support (18 actions) | Support and assistance | Organisational resources |
|  |  |  |  |
| “We have back-up routines. Then we use paper and pen and go back to old-school methods, so to speak.” (Informant 20) | Back-up routines (8 actions) |  |  |
|  |  |  |  |
| “I have a healthcare administrator who works in my area, who also is an IT contact person, and we have daily contact about different matters […]. I get very good help from her regarding IT support and so on.” (Informant 12) | Administrative support (20 actions) |  |  |
|  |  |  |  |
